# The Coronation

**Published:** 1911-06-24

**Authors:** 


### The Hospital

A Journal of

The Medical Sciences and Hospital Administration.

New Series. No. 230, Vol. IX. [No. 1302, Vol. L.] SATURDAY, JUNE 24, 1911

### THE CORONATION.

At a time like the present, when hospital workers and members of the medical profession join with their fellow-citizens in wishing King George a long and prosperous reign, it is permissible to draw attention once more to the close personal interest which the Sovereign has always shown in the welfare of hospitals and charitable institutions in this country. During the year of mourning that has elapsed since the death of the late King it has been proved on more than one occasion that his successor has maintained that close interest in hospitals and their work which characterised his father. The visits to the London and Norfolk Hospitals, which were among the few semi-public functions in which King George was a central figure during the past twelvemonth, and the other instances which we have from time to time reported when the King has personally interested himself in the progress of some institution, have shown clearly that although King George can act no longer as an active member of any hospital board nor as president of an institution, he still, as patron of the hospitals with which he has been associated as Prince of Wales, exercises a powerful and beneficial influence upon hospitals, and, what is of far greater importance to the community, his patronage is a stimulant to personal activity and a guarantee of the excellence of the institutions which are honoured by possessing it. Our hospital system is at the present moment a subject of keen discussion and attention, not only by those who are experienced in the work that is carried on under it, but by the layman who has little or no personal acquaintance with the details and difficulties of that work. At such a time we gratefully realise the fact that His Majesty's support of the work that voluntary hospitals are doing has been a material asset to those upon whom falls the burden of providing for the upkeep and maintenance of these institutions. The knowledge that such support is tendered by one who has himself given personal service on behalf of hospitals, and whose criticism is expert because it is grounded on an intimate acquaintance with the daily routine work of our institutions, is an added source of gratification to everyone connected with hospitals. While the Sovereign, under our constitutional system, stands severely outside party politics and questions at issue between different sections, his influence has been exerted on behalf of all charitable work which knows neither party nor faction but benefits the whole of the community. His example may therefore serve to stimulate us to deal with the hospital problem that confronts us at the present time in an equally detached and independent spirit. Only by attacking it in that fashion can we hope to achieve final results which will strengthen the system and prove of benefit to the nation.

Not only in this country but in every part of the wide Empire over which he rules, King George has subjects who are earnest workers in the field of charity—workers who have been encouraged in the past by his support and who will be cheered in the future by the knowledge that the King is still himself a hospital worker in the best sense of the term. To all of these the Coronation will be the occasion for the expression of a fervent wish that his reign may be a happy, peaceful, and prosperous one!

